# Enhancing medication appropriateness: Insights from the STOPP (Screening Tool of Older Persons’ Prescriptions) criteria version 3 on prescribing practices among the older adults in Pakistan

**DOI:** 10.3389/fphar.2025.1551819

**Published:** 2025-05-20

**Authors:** Halima Sadia, Safila Naveed, Hina Rehman, Shazia Jamshed, Huma Dilshad

**Affiliations:** ^1^ Department of Pharmacy Practice, Faculty of Pharmacy, Jinnah University for Women, Karachi, Pakistan; ^2^ Faculty of Pharmacy and Pharmaceutical Sciences, University of Karachi, Karachi, Pakistan; ^3^ Department of Pharmacy Practice, Institute of Pharmaceutical Sciences, Jinnah Sindh Medical University, Karachi, Pakistan; ^4^ Department of Pharmacy Practice, School of Pharmacy, IMU (Former International Medical University), Kuala Lumpur, Malaysia; ^5^ Department of Pharmacy Practice, Shifa College of Pharmaceutical Sciences, Shifa Tameer-e-Millat University, Islamabad, Pakistan; ^6^ Department of Pharmaceutics, Faculty of Pharmacy, Jinnah University for Women, Karachi, Pakistan

**Keywords:** older adults, Pakistan, STOPP criteria version 3, polypharmacy, multimorbidity

## Abstract

**Background:**

The prevalence of potentially inappropriate medications (PIMs) in older adults populations is a significant concern, often leading to adverse drug events and increased health-care utilization.

**Objective:**

In the present study, we aim to evaluate the prevalence of PIMs among hospitalized older adults patients in Pakistan using STOPP (Screening Tool of Older Persons’ Prescriptions) criteria version 3.

**Methodology:**

A prospective observational study was conducted at a tertiary-care hospital in Karachi over 1 year from March 2023 to March 2024. Patients aged 60 years and above, prescribed at least one medication, were included. Data on demographics, comorbidities, and medications were collected and analyzed using the STOPP criteria to identify PIMs. Statistical analysis was performed using IBM SPSS Statistics version 21. To find the variables linked to PIM use, multivariable logistic regression analysis was used. The 95% CI and adjusted odds ratio (aOR) were used to measure the statistical association’s strength. A p-value of less than 0.05 was deemed statistically significant.

**Results:**

Among 450 participants, the median age was 67 years, with a predominance of male patients (55.3%). The prevalence of PIM use was 56.6%, and a total of 388 instances of PIM use were identified according to STOPP criteria version 3. Acetylsalicylic acid (18%) and pheniramine (11%) were the most frequent inappropriately prescribed medications. The multivariable logistic regression analysis revealed that polypharmacy and the presence of one or more comorbidities primarily influence the PIM use.

**Conclusion:**

The findings highlight a critical need for improved prescribing practices in the older adults population in Pakistan. Utilizing screening tools like the STOPP criteria can significantly enhance medication safety and optimize pharmacotherapy in this vulnerable group.

## Introduction

Potentially inappropriate medication (PIM) use is a significant concern in the older adults population, as it can lead to adverse drug events, hospitalizations, and other negative health outcomes (Lavan and Gallagher, 2016). With the growing age, there is an increased prevalence of chronic diseases which progress to multiple comorbidities. These multiple comorbidities are often accompanied by polypharmacy. The former and the latter are important factors that subsequently lead to the use of potentially inappropriate medication in this vulnerable population (Hudhra et al., 2016; Khatter et al., 2021).

Polypharmacy and PIM go hand in hand. There have been more than 20 definitions proposed for the term polypharmacy, with all of them revolving around the number of medications prescribed to the patient. The numeric threshold of medications for polypharmacy varies from 4 to 10 or even more (Khezrian et al., 2020). Polypharmacy may also be referred to as the use of unnecessary medication having a significant negative impact on patients’ health. Additionally, it also exerts an economical burden through misutilization of health-care services, wastage of limited medical resources, and additional costs to treat any adverse event (Kojima et al., 2012). A number of studies have documented that optimization of pharmacotherapy in the older adults cannot be just achieved by tackling polypharmacy *per se* but also by considering the inappropriate prescribing of medications (Scheen, 2014; Topinková et al., 2012).

The impact and consequences of polypharmacy on patient care are not recent findings and have been known since years. However, it is noteworthy that its complexity and magnitude is increasing day by day. The presence of comorbidities in the older adults warrants the need of polypharmacy; however, its negative impact cannot be neglected (Drusch et al., 2021). It includes but is not limited to medication errors, adverse drug events, medication nonadherence, and drug interactions. Compared to younger individuals, prescribing in the older adults is complex. Owing to age-related organ pathophysiology and declined function of the regulatory processes, the older adults are at an increased risk of inappropriate prescribing outcomes (Fulton and Riley Allen, 2005).

It has been well documented in a number of epidemiological studies that in patients aged 50 years and above, the prevalence of polypharmacy ranges from 12% to 48%, which is quite alarming (Aubert et al., 2016). Identification of the inappropriate medications and medications having higher risks with adverse outcomes is a useful tool to improve prescribing in the older adults. Taking this into account, numerous screening tools have been developed. The most commonly cited tools are the START (Screening Tool to Alert to Right Treatment)/STOPP (Screening Tool of Older Persons Prescriptions), the Medication Appropriateness Index (MAI) (Hanlon and Schmader, 2013), and the Beers Criteria (American Geriatrics Society 2015 Beers Criteria Update Expert Panel, 2015). A concise explanation regarding why the prescribing practice is potentially inappropriate is associated with each criterion.

The START/STOPP tool was first used in 2008, and till date, it is the most widely used and validated tool. It was developed by a panel of United Kingdom and Irish experts. The START criteria identify the medications that are needed to optimize medication therapy in the older adults, while the STOPP criteria identify medications that may impose potential harm in this population (Abukhalil et al., 2022). Recently, the tool has been updated in 2023 (O‘Mahony et al., 2023). The latest version comprises a set of 190 criteria (57 START and 133 STOPP criteria) compared to 114 criteria in version 2 and 87 criteria in version 1. There has been a 66.7% increase in the criteria compared to its predecessors. The majority of the STOPP/START version 3 criteria are based on systematic review and clinical trial evidence.

The START/STOPP criteria have been shown to be more sensitive than Beers criteria in identifying inappropriate prescribing. The tool is easy to use and has several advantages over other tools. The sequential arrangement of the criteria by the physiological systems facilitates its application in everyday practice. Moreover, it also provides a list of potentially inappropriate prescribing (PIPs) by omission (START criteria) (Díaz Planelles et al., 2023). Based on the global population ranking, Pakistan is the sixth most populated country. Presently, the number of older adults is more than eight million, and by 2050, this number is expected to increase more than three times (Cassum et al., 2020). There is a great paucity of data on the magnitude of PIP in Pakistan.

The aim of the present study is to explore the prevalence of inappropriate prescribing in the Pakistani older adults population in light of STOPP criteria version 3 and to explore the factors that influence inappropriate prescribing. In this study, every STOPP criterion was used. The START criteria were not used because the purpose of this study was not to assess possible prescribing omissions issues.

## Methodology

### Study setting and design

A prospective observational study design was implemented in an 850-bed tertiary-care hospital of Karachi, Pakistan, providing a range of medical and surgical services to the residents of Karachi.

All patients aged 60 years and above admitted to the inpatient department of the hospital fulfilling the inclusion criteria, i.e., prescribed with at least one medication, were included in the study. To achieve the maximum number of hospitalized older adults patients, the study was conducted in the medicine, cardiology, nephrology, orthopedic, and surgical wards of the inpatient department. The wards were selected on the basis of the list of the wards provided by the hospital administration. The list was formulated on the basis of greater admissions of geriatric patients in the mentioned wards. Conversely, patients hospitalized for palliative care, acute conditions, terminal illnesses, intensive care, and short-term prognoses were excluded. Moreover, those who had incomplete medical records and were hospitalized for less than 72 h were also not included.

### Study variables

The prevalence of PIMs was the dependent variable of the study, while gender, age groups, number of medications, and comorbidities were the independent variables.

### Sample size calculation

Single population proportion formula was used to obtain the sample size for the study (Pourhoseingholi et al., 2013). The sample size was obtained with a 95% confidence interval (Z statistic corresponding to 95% confidence interval = 1.96) with precision (minimum effect size) of 0.05 and an expected prevalence of 64% (0.64), as reported by Mazhar et al. (2018). The calculated sample size was then adjusted for 20% participant loss (approximate). Hence, the final required sample size was approximately 425 older adults patients. A total of 450 samples were successfully obtained, achieving 105.8% of the intended sample size. Simple random sampling technique was then employed to select the study participants.

$$n=\frac{Z^{2}P\left(1-P\right)}{d^{2}}.$$

### Data collection

Data were collected for a period of 1 year from 1 March 2023 to 1 March 2024. The demographic characteristics of the patients, along with their current and past medical diagnoses, comorbidity burden, current regular prescription medications, laboratory profiles and readings, and any history of drug allergies or intolerances, were thoroughly documented by the research pharmacist in a data abstraction sheet.

The comorbidity burden was measured by the Charlson Comorbidity Index (CCI) score (Charlson et al., 2022). A higher CCI score indicated greater comorbidity burden. The patients were split into three groups: namely, mild patients (those with CCI scores between 1 and 2), moderate patients (those with CCI scores between 3 and 4), and severe patients (those with CCI scores ≥5 and those without any comorbidity). A history of cardiac arrhythmia, peripheral vascular illness, cerebral vasculopathy, ischemic heart disease, or chronic heart failure was considered a cardio- and cerebrovascular disease of comorbidities in CCI. Diabetes mellitus, cancer, cerebrovascular accidents, hypertension, and coronary artery disease were among the comorbidities identified.

All medications prescribed to the patients fulfilling the inclusion criteria were then classified by the pharmaceutical specialties as endorsed by the WHO Anatomical Therapeutic Classification (ATC) system (Organization and Organization, 2000). The medications were classified up to the ATC level 5. Polypharmacy was defined as taking four or more medications.

The collected data were then assessed according to STOPP criteria version 3 to identify PIM (O’Mahony et al., 2023). The STOPP criteria are helpful in providing an explanation for why the medication is potentially inappropriate. The explanations were classified based on the indication of the medication and the medication used in the respective physiological system.

### Statistical analysis

IBM SPSS Statistics version 21 was used to perform the statistical analysis. Demographic characteristics of the study participants were represented as descriptive statistics by calculating the median, interquartile ranges (IQR), and percentages with a 95% confidence interval. The relationship between each independent variable and the dependent variable (PIM) was examined using cross-tabulation in bivariate analysis, and a crude odds ratio was produced. The final multivariable logistic regression model then included variables found in the bivariate analysis with p < 0.25. To find the variables linked to PIMs, multivariable logistic regression analysis was used. The adjusted OR (aOR) and 95% CI were used to gauge the statistical association’s strength. After adjusting for other predictor variables in a model, the aOR shows how changes in one predictor variable affect the likelihood that a response variable will occur. Statistical significance was defined as a p-value less than 0.05.

### Ethical consideration

The ethical approval for the study was obtained from the Institutional Review Board of the Jinnah University for Women (JUW/IERB/PHARM-ARA-013/2023). Similarly, prior approval was also obtained from the tertiary-care hospital where the study was conducted. Informed verbal consent was also obtained from the study participants.

## Results

### Demographics and clinical characteristics of patients

A total of 450 older adults patients were included in the study. The majority of the study population were men (55.3%) (n = 249). The median (IQR) age of the patients was 67 (74–64) years. More than 78% of the patients had a Charlson Comorbidity Index score greater than two. Hypertension and diabetes mellitus were the most prevalent (49.6% and 33.8%) comorbidities. More than 2,800 medications were prescribed to the study participants, with the median (IQR) medication prescribed per patient being six (8–5). Moreover, more than 85% of the patients were prescribed more than four medications (Table 1).

**TABLE 1:** Patient demographics.

Patient characteristics	Total (N = 450)
Gender distribution, n (%)	
Male	249 (55.3)
Female	201 (44.7)
Age distribution, median (IQR) (years)	67 (74–64)
Age-groups (years), n (%)	
60–64	114 (25.3)
65–69	135 (30)
70–74	95 (21.1)
75–79	35 (7.8)
80–84	33 (7.3)
85 and above	38 (8.4)
Total no. of medications prescribed	2,887
Medications prescribed per patient, median (IQR), n (%)	6 (8–5)
<4 medications	91 (11.1)
5–9 medications	309 (68.7)
>10 medications	50 (20.2)
Comorbidity severity, n (%)	
Mild (CCI 1–2)	95 (21.1)
Moderate (3–4)	261 (58)
Severe (CCI ≥5)	94 (20.9)

### Assessment of PIM using STOPP criteria version 3

STOPP criteria version 3 identified 388 PIMs among the study participants (Table 2). More than 50% of the patients had encountered PIM. Of the 255 patients, 63.9% (n = 163) had at least one PIM, followed by 24.3% (n = 62) having two PIMs, 4.5% (n = 22) having three PIMs, and 2% (n = 5) and 1.2% (n = 3) having four and five PIMs, respectively. The prevalence of the PIMs among the study participants, hence, was found to be 56.6%.

**TABLE 2:** Assessment of PIM using STOPP criteria version 3.

Criteria	Total
Indication of medication	
A1. Any drug prescribed without an evidence-based clinical indication	3
A2. Any drug prescribed beyond the recommended duration, where treatment duration is well defined	0
A3. Any duplicate drug class prescription for daily regular use (as distinct from PRN use), e.g., two concurrent NSAIDs, SSRIs, loop diuretics, ACE inhibitors, anticoagulants, antipsychotics, and opioid analgesics (optimization of monotherapy within a single drug class should be observed prior to considering a new agent)	3
Cardiovascular system	
B1. Digoxin for heart failure with preserved systolic ventricular function (no clear evidence of benefit)	0
B2. Verapamil or diltiazem with NYHA class III or IV heart failure (may worsen heart failure with reduced ejection fraction, i.e., HFREF)	2
B3. Beta-blocker in combination with verapamil or diltiazem (risk of heart block)	0
B4. Ventricular rate-limiting drugs, i.e., beta blocker, verapamil, diltiazem, and digoxin with bradycardia (<50/min), type II heart block, or complete heart block (risk of profound hypotension, asystole)	2
B5. Beta-blocker as monotherapy for uncomplicated hypertension, i.e., not associated with angina pectoris, aortic aneurysm, or other conditions where beta-blocker therapy is indicated (no firm evidence of efficacy)	23
B6: Amiodarone as first-line antiarrhythmic therapy in supraventricular tachyarrhythmias (higher risk of major side effects than beta-blockers, digoxin, verapamil, or diltiazem)	1
B7. Loop diuretic as first-line treatment for hypertension unless there is concurrent heart failure requiring diuretic therapy (lack of outcome data for this indication; safer and more effective alternatives available)	12
B8. Loop diuretic for dependent ankle edema without clinical, biochemical evidence, or radiological evidence of heart failure, liver failure, nephrotic syndrome, or renal failure (leg elevation and/or compression hosiery is usually more appropriate)	9
B9. Thiazide diuretic with current significant hypokalemia (i.e., serum K+ < 3.0 mmol/L), hyponatremia (i.e., serum Na+ < 130 mmol/L) hypercalcemia (i.e., corrected serum calcium >2.65 mmol/L), or with a history of gout (hypokalemia, hyponatremia, hypercalcemia, and gout can be precipitated by thiazide diuretic)	0
B10. Loop diuretic for treatment of hypertension with concurrent urinary incontinence (may exacerbate incontinence)	0
B11. Centrally acting antihypertensives (e.g., methyldopa, clonidine, moxonidine, rilmenidine, and guanfacine), unless clear intolerance of or lack of efficacy with other classes of antihypertensives (centrally active antihypertensives are generally less well tolerated by older people than younger people)	0
B12. Angiotensin-converting enzyme (ACE) inhibitors or angiotensin receptor blockers (ARBs) in patients with hyperkalemia, i.e., serum K > 5.5 mmol/L	0
B13. Aldosterone antagonists (e.g., spironolactone and eplerenone) with concurrent potassium-conserving drugs (e.g., ACEIs, ARBs, amiloride, and triamterene) without monitoring of serum potassium (risk of dangerous hyperkalemia, i.e., > 6.0 mmol/L—serum K should be monitored regularly, i.e., at least every 6 months)	1
B14. Phosphodiesterase type-5 inhibitors (e.g., sildenafil, tadalafil, and vardenafil) in severe heart failure characterized by hypotension, i.e., systolic BP < 90 mmHg, or concurrent daily nitrate therapy for angina (risk of cardiovascular collapse)	0
B15: Drugs that predictably prolong the QTc interval (QTc = QT/RR) in patients with known with known QTc prolongation (to >450 msec in males and >470 msec in females), including quinolones, macrolides, ondansetron, citalopram (doses >20 mg/day), escitalopram (doses >10 mg/day), tricyclic antidepressants, lithium, haloperidol, digoxin, class 1A antiarrhythmics, class III antiarrhythmics, tizanidine, phenothiazines, astemizole, and mirabegron (risk of life-threatening ventricular arrhythmias)	6
B16: Statins for primary cardiovascular prevention in persons aged ≥85 years (lack of evidence of efficacy) and established frailty with expected life expectancy less than 3 years	1
B17: Long-term systemic, i.e., non-topical NSAIDs with known history of coronary, cerebral, or peripheral vascular disease (increased risk of thrombosis)	1
B18: Long-term antipsychotics with known history of coronary, cerebral, or peripheral vascular disease (increased risk of thrombosis)	2
B19: NSAIDs or systemic corticosteroids with heart failure requiring loop diuretic therapy (risk of exacerbation of heart failure)	4
B20. Antihypertensive drugs in severe symptomatic aortic stenosis except for RAS inhibitors (risk of severe hypotension and syncope)	0
B21. Digoxin as first-line treatment for long-term (>3 months) ventricular rate control in atrial fibrillation (increased mortality from long-term digoxin use; cardio-selective beta-blockers are generally preferable)	7
Coagulation system	
C1. Long-term aspirin at doses greater than 100 mg per day (increased risk of bleeding and no evidence for increased efficacy)	5
C2. Antiplatelet agents, vitamin K antagonists, direct thrombin inhibitors, or factor Xa inhibitors with concurrent significant bleeding risk, i.e., uncontrolled severe hypertension, bleeding diathesis, and recent nontrivial spontaneous bleeding (high risk of bleeding)	4
C3. Aspirin plus clopidogrel as long-term secondary stroke prevention, i.e., >4 weeks, unless the patient has a coronary stent(s) inserted in the previous 12 months or concurrent acute coronary syndrome or has a high-grade symptomatic carotid arterial stenosis (no evidence of long-term benefit over clopidogrel monotherapy)	4
C4. Antiplatelet agents in combination with vitamin K antagonist, direct thrombin inhibitor, or factor Xa inhibitors in patients with chronic atrial fibrillation, unless there is concurrent coronary artery stent(s) inserted or angiographically proven high-grade (>50%) coronary artery stenosis (no added benefit from antiplatelet agents)	2
C5. Antiplatelet agents with vitamin K antagonist, direct thrombin inhibitor, or factor Xa inhibitors in patients with stable coronary, cerebrovascular, or peripheral arterial disease without a clear indication for anticoagulant therapy (no added benefit from dual therapy)	13
C6. Ticlopidine in any circumstances (clopidogrel and prasugrel have similar efficacy, stronger evidence, and fewer side effects)	0
C7. Antiplatelet agents as alternatives to vitamin K antagonists, direct thrombin inhibitors, or factor Xa inhibitors for stroke prevention in patients with chronic atrial fibrillation (no evidence of efficacy)	0
C8: Vitamin K antagonist, direct thrombin inhibitor, or factor Xa inhibitors for first deep venous thrombosis without continuing provoking risk factors for longer than 6 months (no proven added benefit)	0
C9. Vitamin K antagonist, direct thrombin inhibitor, or factor Xa inhibitors for first pulmonary embolus without continuing provoking risk factors for longer than 6 months (no proven added benefit)	0
C10. NSAIDs and vitamin K antagonist, direct thrombin inhibitor, or factor Xa inhibitors in combination (risk of gastrointestinal bleeding)	3
C11: Vitamin K antagonist as first-line anticoagulant for atrial fibrillation, unless there is concurrent metallic heart valve *in situ*, moderate-to-severe mitral stenosis, or creatinine clearance less than 15mL/min (direct thrombin inhibitor or factor Xa inhibitors are equally efficacious and safer than vitamin K antagonists)	3
C12: Selective serotonin reuptake inhibitors in combination with vitamin K antagonist, direct thrombin inhibitor, or factor Xa inhibitors with a previous history of major hemorrhage (increased risk of bleeding due to antiplatelet effects of SSRIs)	1
C13: Direct thrombin inhibitor (e.g., dabigatran) and diltiazem or verapamil (increased risk of bleeding)	0
C14: Apixaban, dabigatran, edoxaban, rivaroxaban, and P-glycoprotein (P-gp) drug efflux pump inhibitors, e.g., amiodarone, azithromycin, carvedilol, cyclosporin, dronedarone, itraconazole, ketoconazole (systemic), macrolides, quinine, ranolazine, tamoxifen, ticagrelor, and verapamil (increased risk of bleeding)	2
C15: Systemic estrogens or androgens with a pervious history of venous thromboembolism (increased risk of recurrent venous thromboembolism)	0
C16: Aspirin for primary prevention of cardiovascular disease	15
Central nervous system	
D1. Tricyclic antidepressants in patients with dementia, narrow angle glaucoma, cardiac conduction abnormalities, lower urinary tract symptoms related to benign prostatic hyperplasia, chronic constipation, recent falls, or prior history of urinary retention (risk of worsening these conditions)	0
D2. Initiation of tricyclic antidepressants as first-line treatment for major depression (higher risk of adverse drug reactions with TCAs than with SSRIs or SNRIs)	0
D3. Serotonin/noradrenaline reuptake inhibitors (SNRIs, e.g., venlafaxine and duloxetine) and severe hypertension, i.e., systolic blood pressure >180 mmHg ± diastolic blood pressure >105 mmHg (likely to make hypertension worse)	0
D4. Antipsychotics with moderate marked antimuscarinic/anticholinergic effects (acepromazine, chlorpromazine, clozapine, flupenthixol, fluphenazine, levomepromazine, olanzapine, pipotiazine, promazine, and thioridazine) with a history of lower urinary tract symptoms associated with benign prostatic hyperplasia or previous urinary retention (high risk of urinary retention)	0
D5: Antipsychotics prescribed for behavioral and psychological symptoms of dementia (BPSD) at an unchanged dose for >3 months without medication review (increased risk of extrapyramidal side effects and chronic worsening of cognition; increased risk of major cardiovascular morbidity and mortality)	0
D6. Selective serotonin reuptake inhibitors (SSRIs) with current or recent significant hyponatremia, i.e., serum Na+ < 130 mmol/L (risk of exacerbating or precipitating hyponatremia)	0
D7. Selective serotonin re-uptake inhibitors (SSRIs) with current or recent significant bleeding (risk of exacerbation or recurrence of bleeding due to antiplatelet effects of SSRIs)	4
D8. Benzodiazepines for ≥4 weeks (no indication for longer treatment; risk of prolonged sedation, confusion, impaired balance, falls, and road traffic accidents; all benzodiazepines should be withdrawn gradually if taken for >2 weeks as there is a risk of causing a benzodiazepine withdrawal syndrome if stopped abruptly)	7
D9. Benzodiazepines for agitated behavior or noncognitive symptoms of dementia (no evidence of efficacy)	2
D10. Benzodiazepines for insomnia for ≥2 weeks (high risk of dependency, increased risk of falls, fractures, and road traffic accidents)	4
D11. Z-drugs (zolpidem, zopiclone, and zaleplon) for insomnia for ≥2 weeks (increased risk of falls and fractures)	0
D12. Antipsychotics (i.e., other than clozapine or quetiapine) in those with parkinsonism or dementia with Lewy bodies (risk of severe extrapyramidal symptoms)	2
D13. Anticholinergics/antimuscarinic drugs (biperiden, orphenadrine, procyclidine, and trihexyphenidyl) to treat extrapyramidal side effects of antipsychotic medications (risk of anticholinergic toxicity)	7
D14. Drugs with potent anticholinergics/antimuscarinic effects in patients with delirium or dementia (risk of exacerbation of cognitive impairment)	1
D15. Neuroleptic antipsychotics in patients with noncognitive symptoms of dementia (NCSD) for longer than 12 weeks, unless symptoms are severe and other treatments have failed (increased risk of stroke and myocardial infarction)	0
D16. Neuroleptic antipsychotics as hypnotics, unless sleep disorder is due to psychosis or noncognitive symptoms of dementia (risk of confusion, hypotension, extrapyramidal side effects, and falls)	0
D17. Acetylcholinesterase inhibitors with a known history of persistent bradycardia (<60 beats/min.), heart block, or recurrent unexplained syncope (risk of cardiac conduction failure, syncope, and injury)	0
D18. Acetylcholinesterase inhibitors with concurrent treatment with drugs that reduce heart rate such as beta-blockers, digoxin, diltiazem, and verapamil (risk of cardiac conduction failure, syncope, and injury)	0
D19. Memantine with known current or previous seizure disorder (increased risk of seizures)	0
D20. Nootropics in dementia including *Ginkgo biloba*, piracetam, pramiracetam, phenylpiracetam, aniracetam, phosphatidylserine, modafinil, L-theanine, omega-3 fatty acids, *Panax ginseng*, rhodiola, and creatine (no evidence of efficacy)	0
D21. Phenothiazines as first-line treatment for psychosis or noncognitive symptoms of dementia (NCSD) as safer and more efficacious alternatives exist (phenothiazines are sedative and have significant antimuscarinic toxicity in older people, with the exception of prochlorperazine for nausea/vomiting/vertigo, chlorpromazine for relief of persistent hiccups, and levomepromazine as an antiemetic in palliative care)	0
D22. Levodopa or dopamine agonists for benign essential tremor (no evidence of efficacy)	0
D23. Levodopa or dopamine agonists for treatment of extrapyramidal side effects of antipsychotics or other forms of drug-induced Parkinsonism (inappropriate prescribing cascade to be avoided)	0
D24. First-generation antihistamines as first-line treatment for allergy or pruritus (safer, less toxic antihistamines with fewer side effects are now widely available)	44
D25. First-generation antihistamines for insomnia (high risk of side effects; Z-drugs are safer and more appropriate for short-term use)	0
Renal system	
E1. Digoxin at a long-term (i.e., more than 90 days) maintenance dose ≥125 µg/day if eGFR <30 mL/min/1.73 m^2^ (risk of digoxin toxicity if plasma levels not measured)	0
E2. Direct thrombin inhibitors (e.g., dabigatran) if eGFR <30 mL/min/1.73 m^2^ (risk of bleeding)	0
E3. Factor Xa inhibitors (e.g., rivaroxaban and apixaban) if eGFR <15 mL/min/1.73 m^2^ (risk of bleeding)	0
E4. NSAIDs if eGFR <50 mL/min/1.73 m^2^ (risk of deterioration in renal function)	39
E5. Colchicine if eGFR <10 mL/min/1.73 m^2^ (risk of colchicine toxicity)	0
E6. Metformin if eGFR <30 mL/min/1.73 m^2^ (risk of lactic acidosis)	1
E7. Mineralocorticoid receptor antagonists (e.g., spironolactone and eplerenone) if eGFR <30 mL/min/1.73 m^2^ (risk of dangerous hyperkalemia)	0
E8. Nitrofurantoin if eGFR <45 mL/min/1.73 m^2^ (increased risk of nitrofurantoin toxicity)	0
E9. Bisphosphonates if eGFR<30 mL/min/1.73 m^2^ (increased risk of acute renal failure)	0
E10. Methotrexate if eGFR <30 mL/min/1.73 m^2^	0
Gastrointestinal system	
F1. Prochlorperazine or metoclopramide with Parkinsonism (risk of exacerbating Parkinsonian symptoms)	0
F2. PPI for uncomplicated peptic ulcer disease or erosive peptic esophagitis at full therapeutic dosage for >8 weeks (dose reduction, earlier discontinuation, or H2 antagonist maintenance is usually indicated)	5
F3. Drugs likely to cause constipation (e.g., antimuscarinic/anticholinergic drugs, oral iron, opioids, verapamil, and aluminum antacids) in patients with chronic constipation where non-constipating alternatives are appropriate (risk of exacerbation of constipation)	0
F4. Oral elemental iron doses greater than 200 mg daily (e.g., ferrous fumarate >600 mg/day, ferrous sulfate >600 mg/day, and ferrous gluconate >1800 mg/day; no evidence of enhanced iron absorption above these doses)	0
F5. Corticosteroids with a history of peptic ulcer disease or erosive esophagitis (risk of relapse unless proton pump inhibitor is co-prescribed)	0
F6. Antiplatelet or anticoagulant drugs with a history of gastric antral vascular ectasia (GAVE, “watermelon stomach”) (risk of major gastrointestinal bleeding)	3
F7. Antipsychotics with dysphagia (increased risk of aspiration pneumonia)	0
F8. Megestrol acetate to increase appetite (increased risk of thrombosis and death with unproven efficacy)	0
Respiratory system	
G1. Theophylline as monotherapy for COPD (safer, more effective alternative; risk of adverse effects due to narrow therapeutic index)	1
G2. Systemic corticosteroids instead of inhaled corticosteroids for maintenance therapy in moderate-to-severe COPD (unnecessary exposure to long-term side effects of systemic corticosteroids and effective inhaled therapies are available)	29
G3. Long-acting muscarinic antagonists (e.g., tiotropium, aclidinium, umeclidinium, and glycopyrronium) with a history of narrow angle glaucoma (may exacerbate glaucoma) or bladder outflow obstruction (may cause urinary retention)	0
G4. Benzodiazepines with acute or chronic respiratory failure, i.e., pO2 < 8.0 kPa ± pCO2 > 6.5 kPa (risk of exacerbation of respiratory failure)	0
Musculoskeletal system	
H1. Non-COX-2 selective nonsteroidal anti-inflammatory drug (NSAID) with a history of peptic ulcer disease or gastrointestinal bleeding, unless with concurrent PPI or H2 antagonist (risk of peptic ulcer relapse)	0
H2. NSAIDs with severe hypertension, i.e., systolic blood pressure consistently above 170 mmHg and/or diastolic blood pressure consistently above 100 mmHg (risk of exacerbation of hypertension)	1
H3. Long-term use of NSAID (>3 months) for symptom relief of osteoarthritis pain where paracetamol has not been tried (simple analgesics are preferable and usually as effective for pain relief)	0
H4. Long-term corticosteroids (>3 months) as monotherapy for rheumatoid arthritis (risk of systemic corticosteroid side effects)	0
H5. Corticosteroids (other than periodic intra-articular injections for mono-articular pain) for osteoarthritis (risk of systemic corticosteroid side effects)	0
H6. Long-term NSAID or colchicine (>3 months) for prevention of relapses of gout where there is no contraindication to a xanthine-oxidase inhibitor, e.g., allopurinol and febuxostat (xanthine-oxidase inhibitors are the first choice prophylactic drugs in gout)	0
H7. NSAID with concurrent corticosteroids for treatment of arthritis/rheumatism of any kind (increased risk of peptic ulcer disease)	0
H8. Oral bisphosphonates in patients with a history of upper gastrointestinal disease, i.e., dysphagia, esophagitis, gastritis, duodenitis, or peptic ulcer disease, or upper gastrointestinal bleeding (risk of relapse/exacerbation of esophagitis, esophageal ulcer, and esophageal stricture)	0
H9. Long-term opioids for osteoarthritis (lack of evidence of efficacy and increased risk of serious side effects)	0
Urogenital system	
I1. Systemic antimuscarinic drugs in patients with dementia or chronic cognitive impairment (risk of increased confusion and agitation)	0
I2. Systemic antimuscarinic drugs in patients with narrow-angle glaucoma (risk of acute exacerbation of glaucoma)	0
I3. Systemic antimuscarinic drugs with lower urinary tract symptoms associated with benign prostatic hyperplasia and high post-void residual volume, i.e., > 200 mL (uncertain efficacy and increased risk of urinary retention in older men)	0
I4. Systemic antimuscarinic drugs with constipation (risk of exacerbation of constipation)	0
I5. Alpha-1 receptor antagonists other than silodosin (e.g., alfuzosin, doxazosin, indoramin, tamsulosin, and terazosin) with symptomatic orthostatic hypotension or a history of syncope (risk of precipitating recurrent syncope)	1
I6. Mirabegron in labile or severe hypertension (risk of exacerbation of hypertension)	0
I7: Duloxetine with urinary urgency or urge incontinence (duloxetine is indicated in stress incontinence but not in urinary urgency or urge incontinence)	0
I8. Antibiotic use in asymptomatic bacteriuria (no indication for treatment)	5
Endocrine system	
J1. Sulfonylureas with a half-life (e.g., glibenclamide, chlorpropamide, and glimepiride) with type-2 diabetes mellitus (risk of prolonged hypoglycemia)	12
J2. Thiazolidinediones (e.g., rosiglitazone and pioglitazone) in patients with heart failure (risk of exacerbation of heart failure)	6
J3. Nonselective beta-blockers in diabetes mellitus with frequent hypoglycemic episodes (risk of suppressing hypoglycemic symptoms)	0
J4. Sodium glucose co-transporter (SGLT2) inhibitors (e.g., canagliflozin, dapagliflozin, empagliflozin, and ertugliflozin) with symptomatic hypotension (risk of exacerbation of hypotension)	1
J5. Systemic estrogens with a history of breast cancer (increased risk of recurrence)	0
J6. Systemic estrogens with a history of venous thromboembolism (increased risk of recurrence)	0
J7: Menopausal hormone therapy (estrogen plus progestin) with a history of stenotic coronary, cerebral, or peripheral arterial disease (increased risk of acute arterial thrombosis)	0
J8. Systemic estrogens without progestogens in patients with intact uterus (risk of endometrial cancer)	1
J9. Levothyroxine in subclinical hypothyroidism, i.e., normal free T4, elevated TSH but <10 mU/L (no evidence of benefit and risk of iatrogenic thyrotoxicosis)	2
J10. Vasopressin analogs (e.g., desmopressin and vasopressin) for urinary incontinence or urinary frequency (risk of symptomatic hyponatremia)	0
Drug classes that predictably increase falls risk in susceptible older people	
K1. Benzodiazepines in patients with recurrent falls (sedative; may cause reduced sensorium and impair balance)	6
K2. Antipsychotic drugs in patients with recurrent falls (may cause Parkinsonism)	5
K3. Vasodilator drugs in patients with recurrent falls with persistent postural hypotension, i.e., systolic BP drop ≥20 mmHg and/or diastolic BP drop ≥10 mmHg (risk of syncope and falls)	27
K4. Hypnotic Z-drugs, i.e., zopiclone, zolpidem, and zaleplon, in patients with recurrent falls (may cause protracted daytime sedation and ataxia)	0
K5. Antiepileptic drugs in patients with recurrent falls (may impair sensorium and may adversely affect cerebellar function)	7
K6. First-generation antihistamines in patients with recurrent falls (may impair sensorium)	2
K7. Opioids in patients with recurrent falls (may impair sensorium)	1
K8. Antidepressants in patients with recurrent falls (may impair sensorium)	0
K9. Alpha blockers as antihypertensives in patients with recurrent falls (may cause orthostatic hypotension)	0
K10. Alpha blockers for prostatic bladder outflow symptoms, other than silodosin in patients with recurrent falls (may cause orthostatic hypotension)	0
K11. Centrally acting antihypertensives (may impair sensorium and may cause orthostatic hypotension)	0
K12. Antimuscarinics for treatment of overactive bladder or urge incontinence (may impair sensorium)	0
Analgesic drugs	
L1. Use of oral or transdermal strong opioids (morphine, oxycodone, fentanyl, buprenorphine, diamorphine, methadone, tramadol, pethidine, and pentazocine) as first-line therapy for mild pain (WHO analgesic ladder not observed; paracetamol or NSAIDs not prescribed as first-line therapy)	6
L2. Use of regular (as distinct from PRN) opioids without concomitant laxative (risk of severe constipation)	16
L3. Long-acting opioids without short-acting opioids for breakthrough moderate or severe pain (risk of non-control of severe pain)	0
L4. Topical lidocaine (lignocaine) patch for treatment of chronic osteoarthritis pain (no clear-cut evidence of efficacy)	0
L5. Gabapentinoids (e.g., gabapentin and pregabalin) for non-neuropathic pain (lack of evidence of efficacy)	8
L6. Paracetamol at doses ≥3 g/24 h in patients with poor nutritional status, i.e., BMI <18 or chronic liver disease (risk of hepatotoxicity)	0
Antimuscarinic/anticholinergic drug burden	
M1: Concomitant use of two or more drugs with antimuscarinic/anticholinergic properties (e.g., bladder antispasmodics, intestinal antispasmodics, tricyclic antidepressants, first-generation antihistamines, and antipsychotics) (risk of increased antimuscarinic/anticholinergic toxicity)	3
Total	388

**FIGURE 1:**
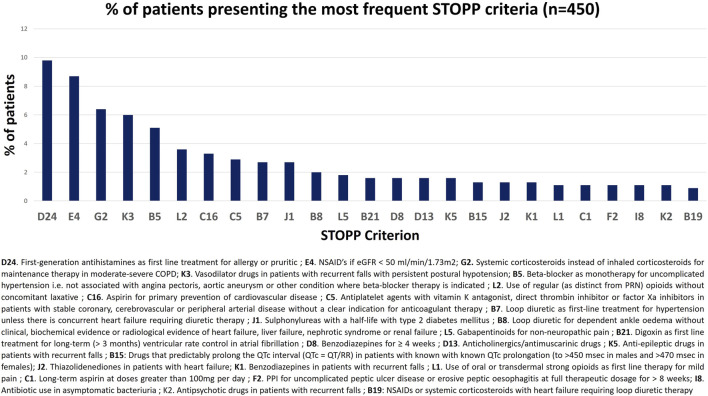
Most commonly identified STOPP criterion.

*First-generation antihistamines as a first-line treatment for allergy or pruritus* was the most frequent PIM (STOPP criterion D24) and was identified in 9.8% of the patients. Subsequently, *NSAIDs (Non-steroidal anti-inflammatory drugs)* if *eGFR <* 50 ml*/min/1.73 m* ^*2*^ *(risk of deterioration in renal function)* (STOPP criterion E4) had the most reported PIM in approximately more than 8% of the patients. Conversely, *opioids in the patients with a history of fall* (criterion K7) and *sodium glucose co-transporter (SGLT2) inhibitors (e.g., empagliflozin) with symptomatic hypotension* (criterion J4) were the least observed. Figure 1 represents the most commonly identified STOPP criteria.

**FIGURE 2:**
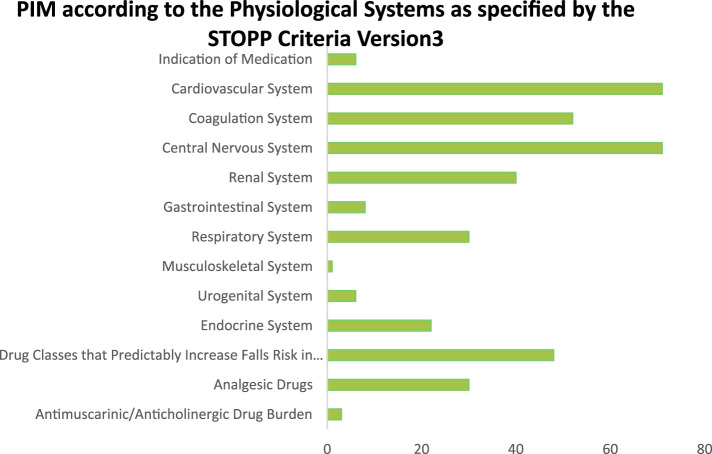
PIM according to the physiological systems.

In terms of PIMs identified by the physiological systems as mentioned in the STOPP criteria, the majority of the PIMs were reported from the cardiovascular system and the central nervous system (18.2%, n = 71), followed by the coagulation system (13.4%, n = 52). Figure 2 illustrates the system-wise occurrence of the PIMs.

Of the total PIMs identified (n = 388), acetylsalicylic acid (B01AC06) (17%) was the most inappropriately prescribed, followed by pheniramine (R06AB05) (11%) and hydrocortisone (H02AB09) (7%). Table 3 highlights the twenty most frequently inappropriately prescribed medications as identified by the STOPP criteria.

**TABLE 3:** Top 20 medications being inappropriately prescribed by the STOPP criteria version 3 (n = 388).

ATC (level 2)	ATC (level 5)	Medication	n	%
A10 Drugs used in diabetes	A10BB12	Glimepiride	8	2
	A10BG03	Pioglitazone	6	2
B01 Antithrombotic agents	B01AA03	Warfarin	12	3
	B01AB05	Enoxaparin	7	2
	B01AC04	Clopidogrel	12	3
	B01AC06	Acetylsalicylic acid	70	18
C01 Cardiac therapy	C01AA05	Digoxin	6	2
	C01DA02	Glyceryl trinitrate	5	1
C02 Antihypertensives	C02DB02	Hydralazine	22	6
C03 Diuretics	C03CA01	Furosemide	27	7
C07 Beta-blocking agents	C07AB03	Atenolol	6	2
	C07AB07	Bisoprolol	5	1
	C07AG02	Carvedilol	7	2
D04 Antipruritics, incl. antihistamines, anesthetics, *etc*.	D04AA16	Pheniramine	44	11
H02 Corticosteroids for systemic use	H02AB09	Hydrocortisone	28	7
M01 Anti-inflammatory and antirheumatic products	M01AB05	Diclofenac	18	5
N02 Analgesics	N02AX02	Tramadol	23	6
N03 Antiepileptics	N03AE01	Clonazepam	10	3
N04 Anti-Parkinson drugs	N04AA04	Procyclidine	9	2
N05 Psycholeptics	N05AX08	Risperidone	6	2

### Factors associated with PIM according to STOPP criteria version 3

A multivariable logistic regression analysis was performed to identify the factors associated with PIM according to STOPP criteria version 3 (Table 4). Among the participants, 54.7% of females and 58.2% of males were found to be using PIMs, and males were 1.31 times more prone to be using PIMs; however, the results were not significantly influenced (p = 0.20).

**TABLE 4:** Multivariable analysis.

Patient characteristics		PIM, n (%)		Crude OR (95% CI)	Adjusted OR (95% CI)	p-value
		Yes	No			
Gender	Female	110 (54.7)	91 (45.3)	1	1	
	Male	145 (58.2)	104 (41.8)	1.15 (0.79–1.77)	1.31 (0.87–1.98)	0.20
Age (years)	60–64	63 (55.3)	51 (44.7)	1	1	
	65–69	77 (57)	58 (43)	1.07 (0.65–1.77)	1.35 (0.77–2.33)	0.29
	70–74	55 (57.9)	40 (42.1)	1.11 (0.64–1.92)	1.38 (0.75–2.53)	0.30
	75–79	22 (62.9)	13 (37.1)	1.37 (1.63–2.99)	1.76 (0.76–4.11)	0.18
	80–84	21 (63.6)	12 (36.4)	1.12 (0.64–3.15)	1.54 (0.66–3.62)	0.32
	85 and above	17 (44.7)	21 (55.3)	0.65 (0.31–1.37)	0.77 (0.35–1.71)	0.53
No. of medications	<4 medications	30 (33)	61 (67)	1		
	5–9 medications	184 (59.5)	125 (40.5)	2.99 (1.82–4.90)	2.89 (1.73–4.81)	0.00^a^
	>10 medications	41 (82)	9 (18)	9.26 (3.98–21.53)	8.42 (3.44–20.63)	0.00^a^
Comorbidities	No comorbidity	53 (38.4)	85 (61.6)	1	1	
	Comorbidity	106 (67.1)	52 (32.9)	3.27 (2.03–5.26)	3.12 (1.89–5.13)	0.00^a^
	Multimorbidity	96 (62.3)	58 (37.7)	2.65 (1.65–4.26)	2.11 (1.27–3.51)	0.00^a^

Under the odd ratios column (crude and adjusted), “1” represents the reference value.

^a^ Significantly associated with the PIMs (<0.05).

Age appeared to have a nuanced relationship with PIMs. Individuals aged 60–79 years showed higher odds of using PIMs, and this was not statistically significant (p = 0.18). In contrast, those aged 85 years and above had a lower likelihood of PIM use (adjusted OR = 0.77), suggesting that older age may not uniformly correlate with increased PIM usage.

A critical finding was the strong association between the number of medications taken and PIM use. Participants on 5–9 medications (adjusted OR = 2.89; 95% CI: 1.73–4.81) and those on more than 10 medications (adjusted OR = 8.42; 95% CI: 3.44–20.63) exhibited even higher odds of PIM use, and both were statistically significant (p < 0.001). This highlights the risk associated with polypharmacy in the older adults.

Similarly, the presence of comorbidities was significantly associated with PIM use. Individuals with comorbidities (adjusted OR = 3.12; 95% CI: 1.89–5.13) and those with multimorbidity (adjusted OR = 2.11; 95% CI: 1.27–3.51) indicated a strong association with the likelihood of being prescribed PIMs (p < 0.001). These results suggest that individuals with one or more comorbidities are significantly more likely to be prescribed PIMs.

## Discussion

The present study is the first study conducted on the Pakistani older adults population to assess the prescription of inappropriate medications and its influencing factors in the older adults population using STOPP criteria version 3. A total of 450 patients aged 60 years and above were included in the study. Based on STOPP criteria version 3, more than 250 of the older adults patients were prescribed inappropriate medications, of which 63.9% (163) had been prescribed at least one PIM.

According to the STOPP criteria used in the study, the prevalence of PIMs in this study (56.6%) is consistent with findings from other research studies conducted in similar settings. For instance, a study by Mazhar et al. reported a prevalence of 64% in a similar demographic, reinforcing the notion that the older adults are particularly vulnerable to inappropriate prescribing practices due to polypharmacy and complex health needs (Mazhar et al., 2018). However, it is noteworthy that the PIMs in this study were identified using explicit STOPP criteria version 2 and the 2015 AGS Beers criteria. The data regarding inappropriate prescribing among the older adults in Pakistan are very scarce, and of the studies reported, very few have employed the STOPP criteria. The majority of the reported studies have employed the AGS Beers criteria (Chang et al., 2023; Sarwar et al., 2018). A study conducted in Larkana reported the prescribing of inappropriate medication in 22.6% of the patients using the 2015 AGS Beers criteria (Mangi et al., 2020). Comparatively, the findings of the present study are also consistent with those of the studies reported in the developed countries. A prospective cross-sectional study in Australia reported PIM prevalence to be 60% (Wahab et al., 2012). Similar prevalence rates were also reported in the studies conducted in Ireland, Brazil, and Israel (Hamilton et al., 2011; Nascimento et al., 2014; Frankenthal et al., 2013). However, some countries have also reported a lower prevalence rate, such as South Korea, Malaysia, and United Kingdom, ranging between 20.5% and 34.5% (Lee et al., 2013; Chen et al., 2012; Gallagher and O’Mahony, 2008). This variability may be attributed due to the presence of more robust systems for medication review, clinical decision support, and pharmacist involvement in medication management in these countries compared to those having high prevalence. This, ultimately, has a significant role in reducing inappropriate prescribing.

The findings of the present study report that 63.9% of the patients had received at least one PIM, which is very high in contrast to a study conducted on cardiac patients to identify PIM using the AGS Beer criteria, which reported that 26.4% patients received at least one PIM (Saqlain et al., 2020). Likewise, approximately 36.5% of the patients had at least one PIM in a study reported by Sarwar et al. The study was conducted in Lahore and, similar to the latter study, the 2015 AGS Beers criteria was used to assess the inappropriate prescribing (Sarwar et al., 2018).

The present finding is also alarming when compared to studies conducted other than in Pakistan, where the prevalence rate was reported to range from 35.4% to 55.4% (Awad and Hanna, 2019; Ryan et al., 2009; Abegaz et al., 2018). These prevalence rates have been evaluated using STOPP criteria version 1 and 2, respectively. This variation may be attributed to the utilization of version 3 of the STOPP criteria for the present study. STOPP criteria version 3 has an extended number of criteria. The comprehensiveness of version 3 is coupled with the evidence-based foundation; hence, it is more sensitive in detecting PIMs than its predecessors (Boland et al., 2023). This is also evident by the only study reported till date regarding the assessment of PIMs by STOPP criteria version 3. Similar to our findings, the study reported the detection of at least one PIM in 74% of the population compared to the 56% detected by version 2 of the STOPP criteria (Szoszkiewicz et al., 2024).

In terms of criterion, the most frequently reported criterion from the STOPP criteria was found to be prescribing of first-generation antihistamines for allergy or pruritus (9.8%). Of the first-generation antihistamines, pheniramine was the most inappropriately prescribed. Due to the strong significant anticholinergic effects of the first-generation antihistamines, they are considered potentially inappropriate and are recommended to be avoided in this vulnerable population by the American Geriatric Association (Cenzer et al., 2020). Moreover, second- and third-generation antihistamines are preferred for the management of allergy or pruritus in the older adults. Pheniramine is a first-generation antihistamine with significant anticholinergic effects. The STOPP criteria explicitly list first-generation antihistamines as PIMs, reflecting the consensus among geriatric experts regarding their risks in older adults patients. The results of the present study are in contrast with a similar study conducted in Taiwan, which reported a lower prevalence of antihistamine prescription in the older adults (Lai et al., 2009). This variability may be likely due to the use of different assessment tools, prescriber’s awareness regarding PIM, and the type of population being studied.

On the basis of physiological systems, the majority of the PIMs were reported from the cardiovascular system and the central nervous system (18.2%, n = 71), followed by the coagulation system (13.4%, n = 52). This finding is consistent with the known risks of medications such as NSAIDs, antiplatelet agents, and antihypertensives in older adults patients with cardiovascular conditions, as well as the risks of anticholinergic drugs and benzodiazepines on cognitive function and fall risk. As a consequence, acetylsalicylic acid was found to be the most frequently inappropriately prescribed medication, followed by R06AB05 pheniramine. The high prevalence of acetylsalicylic acid as a PIM (17%) highlights the need for careful assessment of risk–benefit ratios in older adults patients in Pakistan. Although low-dose aspirin is often used for secondary prevention of cardiovascular events, its use in primary prevention is increasingly questioned, particularly in the older adults due to the risk of bleeding. In Pakistan, where access to advanced diagnostic and monitoring facilities may be limited, the risk of bleeding complications associated with the use of acetylsalicylic acid may be higher. The findings of the present study are consistent with the study reported in Malaysia, Belgium, and Portugal (Fahrni et al., 2019; Dalleur et al., 2012; Candeias et al., 2021). Compared to other studies (Parekh et al., 2019; Monteiro et al., 2020; Zhu et al., 2024), benzodiazepines were not the most common PIM detected; however, clonazepam was found to be in the top ten medications being inappropriately prescribed in the present study.

The multivariable logistic regression analysis identified several independent factors significantly associated with PIM use. Notably, the number of medications prescribed emerged as a strong predictor. Prescribing of five or more medications was strongly associated with the increasing use of PIMs. In this study, over 85% of the participants were prescribed more than four medications, with a median of six medications per patient. This high rate of polypharmacy is consistent with that in previous research, indicating that the older adults often require complex medication regimens due to multiple comorbidities. This is evident by the study conducted in Austria where multimorbidity was the major risk factor for polypharmacy and, as a consequence, polypharmacy emerged in more than 50% of the patients (Schuler et al., 2008). Additionally, the risk of adverse effects is much higher in individuals with polypharmacy than in those prescribed fewer medications (Ahmed et al., 2014). Previous studies have also supported this finding and have emphasized the positive correlation of polypharmacy and PIMs (Steinman et al., 2006; Sayın et al., 2022; MacRae et al., 2021; Bhatt et al., 2019).

It can be a challenging task to prescribe in the older adults considering multiple diagnoses and the deterioration of their physiological conditions (Mangoni and Jackson, 2004; Milton et al., 2008). PIMs cannot be intervened without understanding the influence of comorbidity and multimorbidity on medication prescribing. In the present study, comorbidity and multimorbidity were remarkably associated with the use of PIMs. This is in agreement with the findings reported by Castillo-Páramo et al. (2014).

Although age-groups and gender were not significantly associated with the PIM use, it was found that males were more at risk to be prescribed inappropriate medications than females. This is in contrast to the previous findings where females were more likely to experience PIMs than males (Morgan et al., 2016; Bierman et al., 2007). Moreover, it is widely reported that increasing age is associated with increased risk of PIM (Zhu et al., 2024; Drusch et al., 2021). The same was observed in the present study; however, it was also observed that the risk of PIM use decreases in the individuals aged 85 years and above. Similar findings were reported in a study conducted in the United States (Jirón et al., 2016). Possible explanation for this variability in the present study could be the relatively small sample size in the age bracket of 85 years and above.

## Limitations, strengths, and future directions

The present study provides valuable insights into PIM prevalence and the associated factors among older adults patients in Pakistan. However, it has limitations that warrant consideration. The observational design limits causal inferences regarding the relationships identified between the independent variables and PIM use. Furthermore, conducting the study at a single tertiary-care hospital may limit the generalizability of the findings to other health-care settings or regions within Pakistan.

Nevertheless, the study employed a prospective observational design, allowing for real-time data collection and minimizing recall bias, which is crucial in geriatric research. Furthermore, the use of STOPP criteria version 3 for identifying PIMs ensures that the assessment is based on established and validated guidelines tailored for the older adults. Last but not the least, the present study is the first that prospectively assessed inappropriate prescribing among the older adults in Pakistan using STOPP criteria version 3.

Future research should consider multicenter studies to capture a broader spectrum of prescribing practices across different health-care environments, especially in a country such as Pakistan. Longitudinal studies could also provide insights into how changes in medication regimens over time affect PIM prevalence and patient outcomes.

## Conclusion

The study’s findings highlight the alarmingly high rate of potentially inappropriate prescribing among the older adults population of Karachi, Pakistan. The findings highlight critical factors such as polypharmacy and comorbidity burden that contribute to this problem. Addressing these challenges through improved prescribing practices and systematic medication reviews will be essential for enhancing medication safety and overall health outcomes for the older adults in Pakistan. As the older adults population continues to grow, prioritizing appropriate pharmacotherapy will be crucial in mitigating risks associated with polypharmacy and ensuring better quality care for this vulnerable demographic.

## Data Availability

The original contributions presented in the study are included in the article/supplementary material; further inquiries can be directed to the corresponding author.
